# A Toolkit for Delirium Identification and Promoting Partnerships Between Carers and Nurses: A Pilot Pre–Post Feasibility Study

**DOI:** 10.1007/s11606-024-08734-6

**Published:** 2024-04-22

**Authors:** Christina Aggar, Alison Craswell, Kasia Bail, Roslyn M. Compton, Mark Hughes, Golam Sorwar, James Baker, Jennene Greenhill, Lucy Shinners, Belinda Nichols, Rachel Langheim, Allison Wallis, Karen Bowen, Hazel Bridgett

**Affiliations:** 1 Faculty of Health, Southern Cross University, Bilinga, QLD Australia; 2 School of Health, University of the Sunshine Coast, Maroochydore BC, QLD Australia; 3 College of Nursing, University of Saskatchewan, Saskatoon, SK Canada; 4 Faculty of Health, University of Canberra, Bruce, ACT Australia; 5 Faculty of Business, Law and Arts, Southern Cross University, Bilinga, QLD Australia; 6 Northern NSW Local Health District, Lismore, NSW Australia

**Keywords:** delirium, family, carers, caregiving

## Abstract

**Background:**

Delirium is frightening for people experiencing it and their carers, and it is the most common hospital-acquired complication worldwide. Delirium is associated with higher rates of morbidity, mortality, residential care home admission, dementia, and carer stress and burden, yet strategies to embed the prevention and management of delirium as part of standard hospital care remain challenging. Carers are well placed to recognize subtle changes indicative of delirium, and partner with nurses in the prevention and management of delirium.

**Objective:**

To evaluate a *Pr*evention & *E*arly *Delirium I*dentification *C*arer *T*oolkit (PREDICT), to support partnerships between carers and nurses to prevent and manage delirium.

**Design:**

A pre–post-test intervention and observation study.

**Main Measures:**

Changes in carer knowledge of delirium; beliefs about their role in partnering with nurses and intended and actual use of PREDICT; carer burden and psychological distress. Secondary measures were rates of delirium.

**Participants:**

Participants were carers of Indigenous patients aged 45 years and older and non-Indigenous patients aged 65 years and older.

**Intervention:**

Nurses implemented PREDICT, with a view to provide carers with information about delirium and strategies to address caregiving stress and burden.

**Key Results:**

Participants included 25 carers (43% response rate) (*n* = 17, 68% female) aged 29–88 (*M* = 65, *SD* = 17.7 years). Carer delirium knowledge increased significantly from pre-to-post intervention (*p* =  < .001; CI 2.07–4.73). Carers’ intent and actual use of PREDICT was (*n* = 18, 72%; and *n* = 17, 68%). Carer burden and psychological distress did not significantly change. The incidence of delirium in the intervention ward although not significant, decreased, indicating opportunity for scaling up.

**Conclusion:**

The prevention and management of delirium are imperative for safe and quality care for patients, carers, and staff. Further comprehensive and in-depth research is required to better understand underlying mechanisms of change and explore facets of nursing practice influenced by this innovative approach.

## INTRODUCTION

Delirium, the most common hospital-acquired complication worldwide, is characterized by shifting attention, incoherence, disorientation, and impaired cognition.^1^ It is a frightening experience for the person affected, and their sudden change in behavior and/or emotions can impact family carers’ burden and psychological distress.^1–3^ The global rise in ageing populations is expected to exacerbate the impact of delirium in healthcare settings, leading to increased rates of hospital-acquired complications (e.g., falls), delayed discharge, re-admissions, dementia, residential aged care admissions, death, and greater caregiving responsibilities for families.^4^ Therefore, the prevention, identification, and early management of delirium are imperative in the provision of safe, high-quality care for both the patient and their family.

The healthcare team, including nurses, are responsible for the initial and ongoing assessment, management, and safety of patients at risk of delirium across hospital settings; however, prevention strategies and risk screening are not consistently practiced, and understanding of and recognition of delirium is poor.^5–7^ Reasons for undiagnosed delirium include language barriers, fluctuation of symptoms during the day, a lack of routine screening and assessment, lack of resources, competing clinical priorities, and organization culture.^5,8–13^ These are compounded by a lack of knowledge of the patient’s prior day-to-day level of functioning by the healthcare team.^14^

Rapid deterioration due to delirium begins with subtle changes that are best recognized by family or close ones (referred to as carers here on).^15^ Carers can provide not only a valuable cognitive anchor point but also comforting reassurance, and if supported by clinicians, implement preventative non-pharmacological interventions.^14,16–21^ Interventions implemented with carers to address delirium have been found to improve nurse and carer delirium knowledge,^20^ reduce carer psychological distress,^18,22^ and length of hospital stay.^18,23,24^ However, innovative interventions to support partnerships with carers in the prevention and management of delirium in the hospitalized older patient are needed.^25,26^

### Aim

The primary aim of this study was to evaluate a *Pr*evention & *E*arly *D*elirium *I*dentification *C*arer *T*oolkit (PREDICT) to support partnerships between carers and nurses to prevent and manage delirium. Specifically, the study aimed to evaluate changes in the carer:

•Knowledge of delirium prevention and management
•Beliefs about their role in partnering in delirium prevention and management
•Actual and intended use of PREDICT
•Levels of burden and psychological distress

A secondary aim of this study was to evaluate changes in the incidence of delirium. We hypothesized that the involvement of nurses would improve their understanding of delirium and lead to changes in nursing practice and delirium incidence rates.

## METHOD AND MATERIALS

### Design

A pre–post-test intervention study was conducted on a medical ward in an Australian regional hospital with data collected during admission (pre-intervention) and 4–6 weeks post-discharge (post-intervention). A further observational study to address the secondary aim examined the incidence of delirium during the intervention period compared to the same period 12 months prior.

### The Intervention (PREDICT)

Acknowledging and valuing the insight and lived experience, a model of care utilizing a *Pr*evention & *E*arly *D*elirium *I*dentification *C*arer *T*oolkit (PREDICT) was codesigned and validated by carers whose family members had been hospitalized and for some had experienced delirium, consumers, and healthcare professionals working in the acute care setting.^27^ PREDICT, available on a digital platform and accessed via QR code, included short videos and information on delirium preventive strategies, risk factors, and non-pharmacological interventions to reorientate older adults who experience delirium. To enable carers to express and communicate their concerns about the person being cared for, an interactive psychometrically tested delirium screening questionnaire suitable for informal or untrained carers was also included.^14^ To support carer well-being and address burden and psychological distress, PREDICT also includes information and links to carer resources such as counselling and social prescribing programs (social service programs that provide activities to improve health and well-being).^27^ PREDICT was also made available in hard copy.

### Participants

Participants were carers of Indigenous patients aged 45 years and older and non-Indigenous patients aged 65 years and older. The lower age range for Indigenous patients was set because people who identify as Indigenous Australians are more likely to develop serious medical conditions earlier in life and have a lower life expectancy than non-Indigenous Australians^28^ (Australian Institute of Health and Welfare, 2023).

#### Eligibility

The carer was eligible to receive PREDICT if visiting at the patient’s bedside daily during hospitalization for ≥ 2 days and could communicate in English or with an interpreter. The carer was not eligible to participate if the patient’s hospital stay was less than 48 h, and the patient was receiving end-of-life care or had a diagnosis of advanced dementia and was unable to communicate or interact.

### Procedure

Prior to the implementation of PREDICT (September 2022 to February 2023), nurses received a delirium education session and orientation to PREDICT, and during the study nurses received ongoing weekly briefings from the lead ward nurse for dementia and delirium. This regular communication was to ensure the nursing staff were equipped to answer questions the carer may have regarding PREDICT, the delirium screening questionnaire, and the study evaluation. Posters promoting PREDICT and the study evaluation were placed in strategic areas around the ward, with contact information for further enquiries.

The admitting nurse offered eligible carers access to PREDICT. Carers were advised that they were not required to participate in the study evaluation (that is, complete the study survey) to receive and engage with PREDICT.

Nursing staff were encouraged to support all carers to use PREDICT daily, including the delirium screening questionnaire.^14^ Carers were not offered incentives to participate.

### Data Collection

Participating carers were invited to complete an anonymous survey online using Qualtrics,^29^ or in a paper-based format, at admission (pre-intervention) and 4–6 weeks post-discharge (post-intervention). Pre- and post-intervention surveys were matched using an anonymous participant-generated code (the last 4 digits of participants’ phone numbers, and first initial of their mother’s name). For carers completing a paper-based survey, a secure box was placed at the nurses’ station for surveys returned at admission and a reply-paid envelope for surveys returned at 4–6 weeks follow-up. The incidence of delirium (using the standard unit of measurement of utilization—cases per 1000 occupied bed days (OBDs)) during the intervention period (T2) was compared to the same period 12 months prior (T1).

### Measures

The following measures were combined into the online survey as a continuous tool.

#### Demographics

Carer demographic items included age, gender, whether they identified as Aboriginal or Torres Strait Islander, length of time as a carer, their relationship with the person they cared for, and whether they lived together, as well as the age and gender of the person they cared for.

#### Caregiver Delirium Knowledge Questionnaire (CDKQ)^14^

The CDKQ is a validated measure of carer knowledge of delirium risk factors, symptoms, and appropriate actions with good internal consistency reliability (Cronbach’s *α* = 0.76).^22^ Nineteen true/false items across three subscales include Risk (10 items, e.g., dehydration), Symptoms (5 items, e.g., increasing confusion over several days), and Actions (4 items, e.g., immediately calling a doctor). Total and subscale scores comprise the sum of correct items where higher scores indicate greater knowledge.

#### Beliefs About Carers’ Role in Partnering in Delirium Prevention and Management

A single item question was asked, rated “yes” or “no.”

“Do you think that carers should be incorporated into delirium identification and management?”

#### Carers’ Intended and Actual use of PREDICT, Including the Delirium Screening Questionnaire

Two questions were asked, rated “yes” or “no.”

“Do you intend to use/ Did you use the Delirium Toolkit?”

“Do you intend to use/ Did you use the delirium screening questionnaire?”

#### Caregiver Delirium Burden Scale (DEL-B-C)^30^

The DEL-B-C is a validated 16-item measure of the burden experienced by carers; Cronbach’s *α* = 0.82.^31^ Total scores range from 0 to 40 with higher scores indicating greater burden.

#### Kessler Psychological Distress Scale (K10)^32^

The K10 is a widely used and validated measure of psychological distress; Cronbach’s* α* = 0.93.^32,33^ Total scores range from 10 to 50 with higher scores indicating greater psychological distress.

#### Incidence of Delirium

Routinely collected hospital data was accessed to determine delirium incidence. Data was calculated using cases per 1000 OBDs which were compared from September 2021 to February 2022 (T1) and during the intervention period September 2022–February 2023 (T2).

### Ethical Conduct

Ethical approval was provided by [*removed for blinding*].

### Analysis

Data were entered using Qualtrics survey software,^29^ downloaded and cleaned, checked, and analyzed in SPSS 27^34^ and OpenEpi^35^ analysis software. Summary and descriptive statistics were produced including frequencies, totals, and means of participant demographics and study outcome variables. Significance level was set at alpha (*α*) = 0.05. Normality was established by visual inspection of histograms, skew and kurtosis, and Shapiro–Wilk (as *n* < 50) tests of normality.^36^ Cohen’s *d* effect sizes were calculated as estimates of clinical significance where 0.2 indicates a small effect size, 0.5 moderate, and 0.8 large.^37^ Normally distributed data were assessed for change from admission to post-discharge using paired *t*-tests (CDKQ, DEL-B-C, K10). Non-parametric data were assessed for change using related-samples McNemar change tests for dichotomous dependent variables (beliefs about partnering, satisfaction with care). Relationships between demographics and outcome variables (years as a carer versus intended and actual use of PREDICT) were assessed using independent-samples Mann–Whitney *U* tests. Missing values were handled as follows: frequency data (demographics, beliefs about partnering and use of PREDICT) were unchanged and were reported in raw form; missing CDKQ items were scored as incorrect; missing DEL-B-C items were scored as though carers had not experienced the relevant burden; and no K10 items were missing. Change in delirium incidence was analyzed by calculating an incidence rate ratio (IRR)—that is, comparing incidence at T1 and T2, wherein an IRR of 1 (or 95% CI that includes 1) indicates equal rates of delirium and thus a non-significant change; *Z* (standard) scores and *p* values are also presented for IRRs.^38,39^

## RESULTS

### Participants

PREDICT was provided to a total of 56 carers, of whom 25 carers (43%) provided pre- and post-intervention data. Carers were primarily females (*n* = 17, 68%) with an average age of 65 years (*SD* = 17.7) providing care for their partner (*n* = 15, 60%). The majority of carers (*n* = 17, 68%) lived with the patient prior to admission. A total of seven carers (28%) reported the patient was diagnosed with delirium; see Table 1 for demographics.

**Table 1:** Carer and Patient Demographics

Characteristic	*n* (%)	*M* (*SD*)	Md (IQR)	Range
**Carers**	25 (100)			
Age (years)		65 (17.7)		29 – 88
Gender				
Female	17 (68)			
Male	8 (32)			
Indigenous Australian	1 (4)			
Time as a carer (*years*)			2 (0.4, 5.0)	< 1 – 19
Relationship to patient				
Spouse/partner	15 (60)			
Child	6 (24)			
Other	3 (12)			
Resides with patient	17 (68)			
**Patients receiving care**				
Age (*years*)		77 (9.4)		62 – 89
Gender				
Female	10 (40)			
Male	15 (60)			
Diagnosis of delirium				
Yes	7 (28)			
No	6 (24)			

Values do not sum to 100% due to missing data

*M* mean, *SD* standard deviation, *Md* median, *IQR* interquartile range

### Carer Delirium Knowledge

Carer delirium knowledge (CDKQ)^22^ increased significantly from admission (*M* = 8.7, *SD* = 4.62) to post-discharge (*M* = 12.1, *SD* = 5.43). Scores increased by an average of 3.4 (*SE* = 0.65, 95% CI [2.07, 4.73]; paired *t*(24) = 5.27, *p* =  < 0.001, *d* = 1.1). This very large effect size *(d)* indicates a meaningful, clinically significant improvement in delirium knowledge.^37^

### Beliefs about Partnering in Delirium Prevention and Management

During admission, the majority of carers (*n* = 18, 72%) believed carers should be incorporated into delirium identification and management, which increased to 24 (96%) post-discharge. A related-samples McNemar change test indicated this was a significant increase (*p* < 0.001).

### Carers’ Intended and Actual Use of PREDICT

At admission, most carers intended to use PREDICT (*n* = 18, 72%), and at post-discharge nearly all carers with positive intentions reported they had used PREDICT (*n* = 17, 68%). Similarly, at admission, most carers intended to use the delirium screening questionnaire (*n* = 17, 68%) and at post-discharge most reported they had used it (*n* = 15, 60%), reflecting an effective intention-behavior link with minimal gap;^40^ see Table 2.

**Table 2:** Intended Use of PREDICT and the Delirium Screening Questionnaire During Admission, and Actual Use Post-discharge

Resource	Admission Intended use	Post-discharge Actual use
	***n* (%)**	***n* (%)**
PREDICT		
Yes	18 (72)	17 (68)
No	4 (16)	6 (24)
Delirium screening questionnaire		
Yes	17 (68)	15 (60)
No	4 (16)	7 (28)

Values do not sum to 100% due to missing data

Intended and actual use of PREDICT was associated with total time as a carer, where participants who had been carers for longer were significantly more likely to report intention to use PREDICT (Mann–Whitney *U* = 93.5, *p* = 0.003) and the delirium screening questionnaire (*U* = 90.0, *p* < 0.001) weekly, and actual weekly use PREDICT (*U* = 69.0, *p* = 0.039) and delirium screening questionnaire (*U* = 77.0, *p* = 0.011).

### Carer Burden and Distress

Carer burden (DEL-B-C)^31^ and distress (K10)^32^ did not change significantly from admission to post-discharge (*p* > 0.05). K10 scores were consistently high with carers reporting moderate–high levels of psychological distress at both timepoints^41^; see Table 3.

**Table 3:** Change in Carer Distress and Burden of Delirium from Admission to Post-discharge

Measure	Admission	Post-discharge	Change		Paired-samples *t*-test			
	***M* (*SD*)**	***M* (*SD*)**	***M* (*SE*)**	**95% CI**	***t***	***df***	***p***	***d***
Carer burden of delirium	15.7 (9.19)	17.3 (9.73)	1.6 (1.61)	-1.76, 4.94	0.99	21	0.167	0.21
Kessler Psychological Distress Scale	22.7 (9.14)	23.2 (8.7)	0.5 (0.54)	-0.59, 1.63	0.97	24	0.172	0.19

*M*, mean; *SD*, standard deviation; *SE*, standard error; *CI*, confidence interval

### Incidence of Delirium

The incidence of delirium on the medical ward was 16.6 cases per 1000 OBDs for 2022/2023, compared to 27.1 cases per 1000 OBDs in the 2021/2022 matched period. The incident risk ratio (IRR) for delirium during the period PREDICT was introduced, compared to the same period 12 months prior which was 0.61 [95%CI 0.33, 1.13]. The associated *z*-value was 1.59 with a *p*-value of 0.056 approaching significance. Given the impacts of COVID-19 on healthcare utilization, for contextual comparison points data was also pulled for the whole of hospital, the whole of health district, and the state for the same time periods, which can be seen in Table 4. No other dataset showed any changes in OBD nearing significance, demonstrating promise of association related to the intervention rather than external factors.

**Table 4:** Incidence of Delirium per 1000 Occupied-Bed-Days in the Medical Ward

Setting	Pre-intervention (2021/2022)	Post-intervention (2022/2023)	Incidence risk ratio [95% CI]	*Z*	*p*
Medical ward	27.1	16.6	0.61 [0.33, 1.13]	1.59	0.056
Hospital	6.8	6.6	0.97 [0.33, 2.83]	0.05	0.478
Health district	6.6	6	0.91 [0.3, 2.75]	0.17	0.433
State	5.9	6.2	1.05 [0.34, 3.24]	0.09	0.466

*Incidence*, new delirium diagnoses per 1000 OBDs

## DISCUSSION

There is increasing attention on the importance of the prevention and early management of delirium because of the deleterious effect on older patients’ and carers’ health and well-being.^2,3,30^ This study evaluated the introduction of a model of care utilizing PREDICT, an interactive toolkit designed to support partnerships with carers and nurses in the prevention and management of delirium. The results of this study while only indicative, are promising, highlighting a partnership approach with carers may impact delirium prevention and management.

Several recent systematic reviews and meta-analyses highlight the importance of carer involvement in delirium management^42^ and the efficacy of education;^11,18^ however, many key studies omit the carer perspective.^15^ While most carers in this study significantly increased their knowledge of delirium, we were also able to demonstrate that they saw a clear role for their ongoing involvement in preventing and managing delirium, particularly carers who had been caring for a longer time. This is important because it presents opportunities for improved long-term patient outcomes as the carer is likely to continue to monitor delirium risk following discharge. These findings respond directly to the expectations of carer involvement in care decisions and delivery, as demonstrated by delirium guidelines and standards worldwide.^43–45^

Despite increases in delirium knowledge and the utilization of acquired learnings, carers’ moderately high levels of psychological distress and burden did not significantly improve, contrasting with other studies.^11,22^ While this finding could be due to differences in characteristics of sample populations, it is consistent with studies reporting carers were often highly distressed when the person they were caring for experienced delirium or was at risk of delirium.^46,47^ Perception of burden is multifaceted and changes over time, raising questions as to whether equity measurements, such as social needs and barriers to care, such as transportation, food insecurity, and housing, are more relevant outcome indicators of burden for carers.^48^ While the focus on partnering with carers in our study maximizes the opportunity for enhanced communication and collaboration between carers and nurses, further research is required to elicit the impact of psychological distress and burden in the management of delirium.^30,49^ Where health inequities impact vulnerable groups including LGBTQ + and Indigenous communities,^50^ further research is required to enable carers to highlight their well-being and support needs.

Finally, in relation to our secondary aim, our findings indicate the potential of partnering with carers in delirium prevention and the broad promotion of PREDICT for reducing the incidence of delirium. Given change in the incidence of delirium was not seen elsewhere in the hospital, local health district, or state figures, it is reasonable to hypothesize that PREDICT might have had a ripple effect at the ward level and improved nurses’ delirium prevention practice. Combined with the pre–post-intervention results, there appears to be merit in proceeding to a randomized controlled trial to further validate and understand this model of care and PREDICT’s broader impact.

When deploying this toolkit in additional facilities, it would be of benefit to specifically explore changes in nurse delirium knowledge levels and self-rated confidence in detecting delirium. This would enable improved measurement of the program’s impact on nurses’ understanding and competence in managing delirium cases. It would also be of benefit to include qualitative interviews to better understand how consciously or unconsciously the program may have influenced their practice, altered perceptions of patient interactions, and transformed their overall approach to care, providing a deeper understanding of any mechanisms of change. Finally, future studies could examine any changes to the way in which nurses work when acting in the role of partner in care, including if there are any changes in shared vigilance, improved communication with carers, or changes in intervention strategies. Understanding any mechanisms of change would be crucial for refining program design and understanding its impact.

### Study Limitations

A limitation of this study lies in the small sample size and its location in a single medical ward in an Australian regional hospital. This study did not calculate average length of stay; however, older persons’ hospital service utilization in Australia is reported to average 7.1 days.^51^ A further limitation was that PREDICT was validated with carers in the community^27^ but not an inpatient setting. Finally, PREDICT is limited to those patients who have carers visit at the bedside. While carers are not always at the bedside 24/7, the provision of PREDICT to carers upon admission will support any non-face to face communication between healthcare professionals and carers about the cognitive status of the patient. Future rigorous research as to whether partnering with carers in the prevention of delirium using PREDICT can reduce the incidence of delirium will be an important next step.

## CONCLUSION

This study focused on engaging and supporting carers as partners in the prevention and management of delirium. While this study presents encouraging preliminary results, more extensive research is required seeking to better understand underlying mechanisms of change and exploring additional facets of nursing practice influenced by this innovative approach.

## Data Availability

The data presented in this study are available on request from the corresponding author (Christina Aggar).
